# The neurobehavioral correlates of error processing in adult attention-deficit/hyperactivity disorder and their relationship with impulsivity

**DOI:** 10.1016/j.cnp.2025.11.005

**Published:** 2025-12-02

**Authors:** Gabriele Diamanti, Julien Colin, Roland Hasler, Tomas Ros, Nader Perroud, Marie-Pierre Deiber

**Affiliations:** a Faculty of Medicine, University of Geneva, Geneva, Switzerland; b General Internal Medicine Department, Geneva University Hospitals, Geneva, Switzerland; c Department of Clinical Neurosciences, University of Geneva, Geneva, Switzerland; d CIBM Center for Biomedical Imaging, Geneva-Lausanne, Switzerland; e Division of Institutional Measures, Department of Psychiatry, Geneva University Hospitals, Geneva, Switzerland; f Department of Psychiatry, University of Geneva, Geneva, Switzerland; g Division of Adult Psychiatry, Department of Psychiatry, Geneva University Hospitals, Geneva, Switzerland

**Keywords:** Adult ADHD, Error processing, Event-Related Potentials (ERPs), Impulsivity

## Abstract

**Objective:**

Individuals with Attention-Deficit/Hyperactivity Disorder (ADHD) face stigma in a society that values efficiency and self-control, with their behaviors often misattributed to inattention or recklessness rather than neurophysiological mechanisms. This study examined error processing deficits in adults with ADHD using event-related potentials (ERPs).

**Methods:**

Twenty-eight adults with ADHD and 22 healthy controls completed a Continuous Performance Task (CPT) while undergoing EEG recording. ERP analysis focused on error-related negativity (Ne/ERN), linked to automatic error detection, and error positivity (Pe), associated with conscious error evaluation.

**Results:**

Although reaction times did not differ significantly, ADHD patients showed greater response variability, increased omission and commission errors, and reduced stimulus detectability. After adjusting for anxiety, depression, and the number of valid trials, Ne amplitude did not differ between groups, whereas individuals with ADHD showed significantly reduced Pe amplitude. Within the ADHD group, prolonged Ne latency correlated with increased commission errors and faster responses, while lower Pe amplitude was linked to more commission errors and higher reaction time variability. Ne amplitude was negatively associated with motor impulsivity.

**Conclusions:**

These findings highlight primary deficits in conscious error-monitoring processes, shedding light on altered neurophysiological mechanisms underlying impulsivity in adult ADHD.

**Significance:**

This study provides insights into ADHD-related impulsivity, potentially informing future interventions for executive dysfunction.

## Highlights

• Elevated detection deficits and errors in Attention-Deficit/Hyperactivity Disorder (ADHD)
• Reduced error positivity (Pe) amplitude reflects impaired error awareness in ADHD.
• Altered error negativity (Ne) and positivity (Pe) correlate with impulsivity in ADHD.

## 1 Introduction

Attention deficit hyperactivity disorder (ADHD), once widely misunderstood and stigmatized, is now increasingly recognized as a complex neurodevelopmental condition, thanks to advances in neuroscience and growing public awareness. ADHD typically emerges in childhood and affects between 2 % and 7 % of children (Sayal et al., 2018) and approximately 3.10 % of adults worldwide (Ayano et al., 2023). It is characterized by three core symptom domains: inattention, hyperactivity, and impulsivity (Bader and Perroud, 2012, Weibel et al., 2020), with hyperactivity and impulsivity often declining with age (Krause et al., 2006).

Adults with ADHD frequently experience impairments in attention regulation and impulse control, which can significantly disrupt academic performance, occupational success, and social relationships (Arnsten, 2009, Weibel et al., 2020). Among these symptoms, impulsivity is particularly consequential. It can manifest in difficulties maintaining close interpersonal relationships (Barkley et al., 2006), increased engagement in risky behaviors—including those that elevate the risk of sexually transmitted infections (Soldati et al., 2024)—and a heightened vulnerability to accidental injuries, contributing to elevated mortality rates from non-natural causes (Dalsgaard et al., 2015). Moreover, ADHD is markedly overrepresented in prison populations, with prevalence rates up to five times higher than in the general population (Baggio et al., 2018). These findings underscore the personal and societal burden of impulsivity, highlighting the critical need to understand its underlying neurocognitive mechanisms.

At the neurobiological level, ADHD is linked to disruptions in the brain’s executive system, particularly within the prefrontal cortex (PFC) and anterior cingulate cortex (ACC) (Arnsten, 2009). The PFC is crucial for sustaining attention, inhibiting inappropriate responses, and managing working memory, while the ACC is involved in error detection, conflict monitoring, and emotional regulation (Menon and D'Esposito, 2022). Dysfunction in these regions leads to behavioral disinhibition and frequent errors, which reflect impaired suppression of inappropriate actions or thoughts and serve as measurable indicators of executive control deficits (Arnsten, 2009, Rubia et al., 2005).

This study investigated the neural mechanisms underlying error monitoring to identify their specific alterations in ADHD. Error monitoring is typically assessed using event-related potentials (ERPs), specifically the error-related negativity (ERN or Ne) and the error positivity (Pe). The Ne arises shortly after an erroneous response and is thought to reflect automatic error detection, while the Pe occurs later and is associated with conscious error awareness and post-error adjustment (Falkenstein et al., 2000, Gehring et al., 1993, Steinhauser and Yeung, 2010). Neurophysiological studies consistently show that adults with ADHD exhibit reduced Ne and Pe amplitudes, indicating deficits in both early and late stages of error processing (Balogh et al., 2017, Bellato et al., 2021, Kaiser et al., 2020, Marquardt et al., 2018). These ERP abnormalities may be linked to diminished task performance and increased symptom severity (Devor et al., 2025), and appear to be further modulated by emotional context (Balogh et al., 2017, Figuracion et al., 2024).

The present study aims to characterize Ne and Pe amplitudes and latencies in adults diagnosed with ADHD compared to typically developing controls. Additionally, it examines how these neural markers relate to clinical symptoms and behavioral performance. By doing so, we seek to clarify the neurophysiological profile of error monitoring deficits in ADHD and their relevance to impulsivity and executive dysfunction.

## 2 Materials and methods

This section has been partly described elsewhere (Deiber et al., 2021, Deiber et al., 2020).

### 2.1 Participants

Twenty-eight adult patients with ADHD (15 female, mean age: 34.25, SD: 10.5) were recruited in a specialized center for the assessment, treatment and care of patients suffering from ADHD at the Department of Psychiatry of the University Hospitals of Geneva. At the time of recruitment (usually several months after the initial contact with our center), 10 patients were unmedicated, 11 were taking methylphenidate, 3 atomoxetine, 1 antiepileptic, 1 benzodiazepine, 1 neuroleptic and 1 SNRI (duloxetine). Patients with comorbid psychiatric conditions were excluded. Twenty-two healthy adults (Controls, 14 female, mean age: 31.1, SD: 7.4) were additionally recruited through announcements in the general population. Mean age between groups did not differ significantly (*ANOVA,* F = 1.24, p = 0.271). Prior to the study, written informed consent was obtained from each participant. The study was approved by the Research Ethic Committee of the Republic and Canton of Geneva [project number 2017–01029] (Deiber et al., 2020).

During a first clinical visit, patients and controls underwent three clinical questionnaires: (i) the ADHD Child Evaluation for Adults (ACE + ), a semi-structured interview developed to support healthcare practitioners in the assessment and diagnosis of adults with ADHD (freely available at: https://www.psychology-services.uk.com/adhd), (ii) the French version of the Structured Clinical Interview for DSM-IV Axis II Personality Disorders (SCID-II, (First et al., 1997)) and (iii) the French version of the Diagnostic Interview for Genetic Studies (DIGS, mood disorder parts only (Preisig et al., 1999)). Additionally, all participants completed a number of standardized self-questionnaires to assess ADHD symptomatology. The Adult ADHD Self-Report Scale (ASRS v1.1) evaluates in 18 questions current ADHD symptoms in adolescents and adults (Kessler et al., 2005). The Wender-Utah Rating Scale (WURS) short version (25 items) completes the ASRS to evaluate ADHD symptoms during childhood (Ward et al., 1993). The Barratt Impulsiveness Scale (BIS-11) explores 3 dimensions of impulsiveness: attentional, motor and nonplanning (Patton et al., 1995). Measures of anxiety and depression were additionally administered to assess overall psychological strain. The State-Trait Anxiety Inventory (STAI) is a psychological assessment consisting of 40 self-reported questions on a 4-point Likert scale, designed to measure both state and trait anxiety (Spielberger et al., 1983). The Beck Depression Inventory (BDI) is a widely used self-report assessment with 21 multiple-choice questions for evaluating the severity of depression (Wang and Gorenstein, 2013). The test scores relative to ADHD evaluation are reported in Table 1.

**Table 1:** Demographic Variables and Clinical Scores.

		ADHD			CTL			F(1,48)		*p-value*
Age		34.25	*10.50*		31.32	*7.33*		1.24		0.271
Gender: M / F		13 / 15			8 / 14			0.50		0.484
ACE +: Child attention		7.93	*1.59*		0.05	*0.21*		534.06		<0.001
ACE +: Adult attention		8.00	*1.54*		0.09	*0.43*		545.46		<0.001
ACE +: Child hyperactivity		6.11	*2.82*		0.23	*0.87*		88.68		<0.001
ACE +: Adult hyperactivity		6.11	*2.87*		0.23	*0.69*		87.92		<0.001
ASRS Attention		25.64	*5.15*		10.50	*4.09*		126.91		<0.001
ASRS Hyperactivity		20.86	*6.73*		9.39	*5.22*		43.40		<0.001
WURS		51.54	*17.59*		18.10	*13.15*		55.18		<0.001
BIS: Attentional		22.25	*3.64*		14.45	*2.56*		72.51		<0.001
BIS: Motor		25.22	*5.39*		18.91	*2.35*		26.14		<0.001
BIS: Planning		30.17	*3.72*		20.57	*4.02*		76.36		<0.001
BIS: Total		77.63	*10.58*		53.94	*7.45*		79.22		<0.001
STAI: state		43.05	*15.01*		31.45	*7.34*		11.03		0.002
STAI: trait		53.76	*11.01*		35.25	*7.66*		45.04		<0.001
BDI		17.75	*11.87*		4.45	*4.43*		24.79		<0.001

Mean scores and standard deviations (in italics) for ADHD questionnaire results in ADHD patients (N = 28) and healthy controls (CTL, N = 22), along with one way-ANOVA results. ACE +: ADHD Child Evaluation for Adults, ASRS: ADHD Self-Report Scale, WURS: Wender-Utah Rating Scale, BIS: Barratt Impulsiveness Scale. STAI: State-Trait Anxiety Inventory, BDI: Beck Depression Inventory.

Participants with ADHD exhibited higher levels of anxiety and depressive symptoms compared to controls. However, the measures employed—STAI and BDI—are self-report instruments designed to assess psychological distress, not diagnose psychiatric disorders. All participants were systematically screened in accordance with DSM-5 criteria to exclude current comorbid psychiatric conditions, ensuring the absence of active diagnoses. Importantly, anxiety and depression scores were included as covariates in the statistical analysis to account for their potential impact on ERP measures.

All participants demonstrated strong motivation to take part in the study. In particular, many of the patients had been awaiting access to specialized ADHD services for years, which likely enhanced their engagement and dedication. Visual acuity was verified through inclusion criteria stipulating normal or corrected-to-normal vision, and the visual task stimuli were calibrated with appropriate size and contrast to ensure clear perception and eliminate any visual constraints. Exclusion criteria included: history of head injury with loss of consciousness, epilepsy or stroke, non-neurological conditions susceptible to impair brain function (e.g., cancer or cardiovascular disease), and other current psychiatric disorders based on the above mentioned semi-structured interviews: major depressive disorder, bipolar disorder, anxiety disorders, personality disorder, and substance use disorders. All patients treated with psychostimulants stopped their medication 24 h before the experimental visit. Among the 28 patients, 20 were of the combined presentation, 7 of the predominantly inattentive presentation, and 1 of the predominantly hyperactive-impulsive presentation.

### 2.2 EEG acquisition

Participants were seated approximately 80 cm from a computer monitor in a sound-attenuated, semi-darkened Faraday room to minimize environmental interference during EEG recording. The EEG was recorded continuously using a 64-channel Ag/AgCl electrode cap based on the 10–20 international system, with a sampling rate of 500 Hz. The ground electrode was positioned on the scalp at a site equidistant between Fpz and Fz, and the reference electrode at CPz. Bipolar derivations Fp1-M1 and AF7-AF8 were utilized for detecting vertical and horizontal eye movements, respectively. The electrical signals were amplified using the eego mylab system from ANT Neuro in the Netherlands (https://www.ant-neuro.com/), and all electrode impedances were maintained below 5 kΩ. For offline analyses, EEG signals were re-referenced to a common-average reference. Letter stimuli, response presses as well as feedback codes were automatically marked in the continuous EEG file, and later used to segment the continuous EEG data into epochs time-locked to the event of interest.

The Continuous Performance Task (CPT) employed a Go/NoGo paradigm involving the sequential presentation of 16 letters. Each letter was displayed centrally on the screen for 200 ms. Participants were instructed to press the left mouse button in response to every letter except the target letter “X”. In total, 240 trials were presented, comprising 180 Go trials and 60 NoGo trials. Stimuli were followed by a maximum response window of 600 ms. Intertrial intervals varied pseudo-randomly between 800, 900, and 1000 ms to reduce temporal anticipation.

### 2.3 Data analysis

#### 2.3.1 Behavioral performance during CPT

Reaction time (RT) was defined as the interval between stimulus onset and the corresponding button press. Additionally, the RT variation coefficient was analyzed, calculated as the standard deviation of RT divided by its mean. Errors included omissions and commissions. D-prime was calculated as the ratio between hits and commissions, providing a measure of the ability to discriminate between stimuli. A multivariate analysis of variance was used to analyze the effect of group (ADHD vs. Controls) on behavioral parameters. The significance threshold was set at p < 0.05 after Bonferroni correction for multiple comparisons.

#### 2.3.2 Error-related event-related potentials (ERPs)

The EEG analysis was conducted using the Brain Analyzer 2.0 (Brain Products GmbH, https://www.brainproducts.com/solutions/analyzer/). Ocular movements were corrected using the method of Gratton et al. (Gratton et al., 1983). The data was filtered in the bandwidth of 0.1 to 30 Hz. Segmentation was based on response type, determined by an internal feedback code, within a time window of −800 to 800 ms. The reference time point (0 ms) corresponds to the feedback code, recorded 20 ms after the mouse button press. A linear detrend of the signal was carried out between −800 and 800 ms, based on 100 ms intervals at the beginning and end of the period. Artifact rejection was based on the following criteria: potential variations greater than 50 µvolts/ms; maximum potential difference greater than 150 µvolts over 200 ms intervals; maximum amplitude values greater than 150 µvolts, minimum values less than −150 µvolts over the total time range; amplitude less than 0.5 µvolt over 100 ms intervals. Baseline correction was performed between −200 and −100 ms. An intra-individual average was computed separately for each feedback code type, resulting in distinct average evoked potentials for correct responses (hits) and errors (commissions) in motor-response trials. Inter-individual averages of evoked potentials for correct responses and errors were computed separately for each group.

The Ne and Pe components were derived from the difference waveform between error and correct trials (Manoach and Agam, 2013, Pailing et al., 2002). This subtraction method eliminates shared components between correct and incorrect responses, allowing the resulting waveform to more specifically reflect error-related neural activity rather than task uncertainty (Pailing et al., 2002).

Each participant completed the CPT twice at 40-minute intervals (before and after a neurofeedback session for a separate study). To improve signal-to-noise ratio, trials from both sessions were averaged, effectively doubling the usable data. Crucially, Wilcoxon matched-pair tests revealed no significant differences in Ne and Pe peak amplitudes or latencies between CPT sessions 1 and 2, supporting the validity of this averaging approach.

On average, ADHD participants contributed 33 ± 14 error trials and 345 ± 18 correct trials (totaling 378 ± 15 trials), while controls contributed 21 ± 14 error trials and 355 ± 8 correct trials (totaling 376 ± 14 trials). Although ADHD participants made significantly more errors than controls, there was no significant difference in the total number of trials used to compute the error–correct difference waveform across groups (Mann–Whitney *U* test, p = 0.506). Notably, Ne and Pe were extracted from this difference waveform, which leverages both trial types to optimize component specificity and signal reliability.

Each component was identified in the grand average difference waveform and analyzed at the sites and latencies of their peak activity (Luck, 2014). Hence, Ne was scored at median frontocentral (FC1, FCz, FC2) and central (C1, Cz, C2) electrodes, between –20 and 100 ms around time point 0. Pe was scored at median central (C1, Cz, C2) and centroparietal (CP1, CPz, CP2) electrodes, between 60 and 500 ms following time-point 0.

To assess group effects (ADHD vs. Controls), a multivariate analysis of variance was conducted on Ne and Pe peak amplitudes and latencies. Anxiety (STAI-state) and depression (BDI) scores were included as covariates, along with the average number of trials contributing to each participant’s error–correct difference waveform, to account for potential variance in signal quality. The significance threshold was set at *p* < 0.05, Bonferroni-corrected for multiple comparisons.

#### 2.3.3 Correlation analyses between ERP components and clinical and behavioral scores in ADHD

Spearman correlations were calculated to examine the relationships between Ne and Pe components and the behavioral performance of the participants with ADHD. This included examining the amplitude and latency of Ne and Pe versus RT, RT variation coefficient, omissions, and commissions.

Similarly, the relationship between the Ne and Pe components and the three main clinical impulsivity scores was examined. Spearman correlations were calculated for Ne and Pe amplitude and latency with the adult ACE + hyperactivity score, the ASRS hyperactivity score, and the BIS motor impulsivity score. Significance level was set at p < 0.05 after Bonferroni correction for multiple comparisons.

## 3 Results

### 3.1 Intergroup comparison of CPT performance metrics

Table 2 presents the average values and the comparison of behavioral parameters between groups. While RT did not differ significantly between the two groups, the ADHD group showed a markedly higher RT variation coefficient than the control group. Additionally, individuals in the ADHD group committed significantly more omission and commission errors and demonstrated lower stimulus detectability compared to the control group.

**Table 2:** Behavioral Measures During CPT.

		ADHD			CTL			F(5,44)			*p-value*
Detectability (d’)		2.84	*0.19*		3.70	*0.20*		9.96			0.003
Omission (%)		3.35	*0.62*		0.43	*0.70*		9.61			0.003
Commission (%)		28.21	*2.18*		16.44	*2.46*		12.82			<0.001
Reaction time (ms)		331.1	*7.3*		340.9	*8.3*		0.79			0.378
Variation coefficient (%)		24.92	*0.65*		19.49	*0.73*		30.80			<0.001

Adjusted means and standard errors (in italics) of behavioral parameters, along with multivariate analysis results.

### 3.2 Intergroup comparison of error-related ERPs

**Fig. 1:**
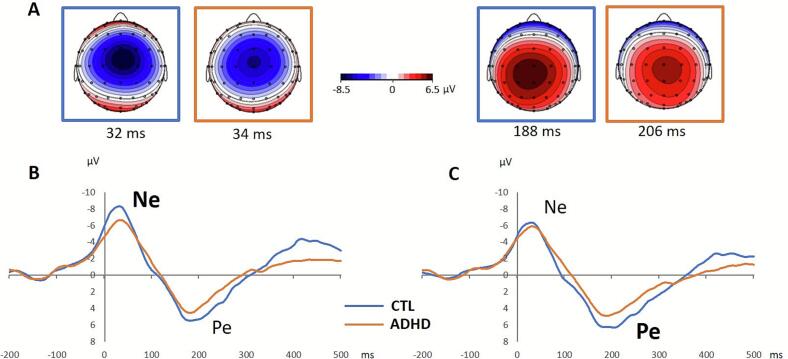
(A) Topographical distribution of Ne and Pe components, and averaged ERPs over (B) frontocentral channels (FC1, FCz, FC2, C1, Cz, C2) and (C) centroparietal channels (C1, Cz, C2, CP1, CPz, CP2), presented for the ADHD group (orange) and control group (blue). (For interpretation of the references to colour in this figure legend, the reader is referred to the web version of this article.)

The Ne and Pe components demonstrated a similar central distribution across both groups, with Ne exhibiting a frontocentral tendency and Pe showing a centroposterior distribution (Fig. 1).

**Fig. 2:**
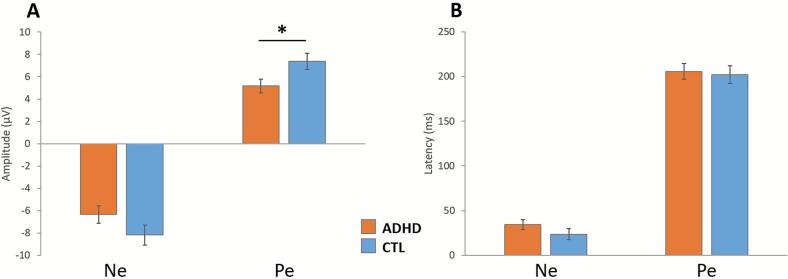
Adjusted mean amplitudes (A) and latencies (B) of Ne and Pe components, with standard errors, for each group.*: *p* < 0.05 (Bonferroni corrected).

Table 3 reports the average Ne and Pe values and their comparison across groups, adjusted for anxiety and depression scores, as well as the average number of valid trials. Fig. 2 displays the mean amplitudes and latencies of both components. Ne amplitude did not differ significantly between the two groups. However, Pe amplitude showed a significant difference, with the ADHD group showing a less pronounced response than the control group. No significant group differences were observed for Ne or Pe latencies.

**Table 3:** Electrophysiological Measures During CPT.

		ADHD			CTL			F(4,45)		*p-value*
Ne peak amplitude (µV)		−6.34	*0.78*		−8.17	*0.90*		1.97		0.167
Ne latency (ms)		34.2	*5.3*		23.5	*6.2*		1.43		0.238
Pe peak amplitude (µV)		5.18	*0.62*		7.38	*0.72*		4.51		0.04*
Pe latency (ms)		205.7	*8.8*		202.0	*10.2*		0.06		0.807

Adjusted means and standard errors (in italics) of Ne and Pe amplitudes and latencies, along with multivariate analysis results adjusted for anxiety, depression, and average number of valid trials. *:significant at p < 0.05 after Bonferroni correction.

### 3.3 Associations between error-related brain responses (Ne, Pe) and behavioral performance (CPT) in ADHD

**Fig. 3:**
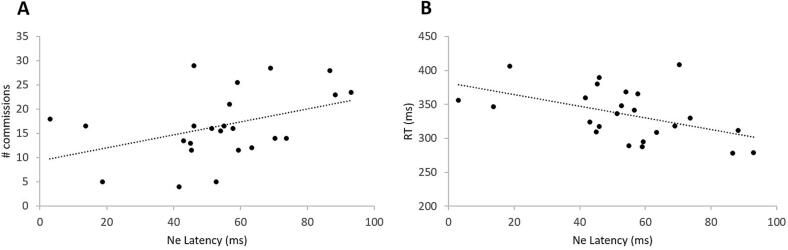
Significant associations within the ADHD group between Ne latency at frontocentral sites and (A) the number of commission errors and (B) the reaction time.

Error-related Ne and Pe responses in ADHD participants were correlated with CPT scores reflecting impulsivity. Analysis of Ne latency revealed a positive correlation with the number of commission errors (Spearman Rho = 0.455, p = 0.015) and a negative correlation with RT (Spearman Rho = -0.482, p = 0.009). Thus, longer Ne latency was associated with a higher number of commission errors (Fig. 3A) and faster RTs (Fig. 3B).

**Fig. 4:**
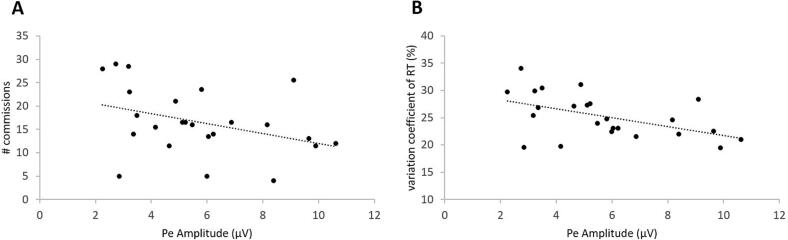
Significant associations within the ADHD group between Pe amplitude at centroparietal sites and (A) the number of commission errors and (B) the variation coefficient of the reaction time.

Statistical analysis identified a negative correlation between Pe amplitude and both the number of commission errors (Spearman Rho = -0.475, p = 0.011) and the RT variation coefficient (Spearman Rho = -0.507, p = 0.006). Accordingly, lower Pe amplitude was linked to a higher number of commission errors (Fig. 4A) and increased RT variability (Fig. 4B).

### 3.4 Associations between error-related brain responses (Ne, Pe) and impulsivity scores in ADHD

**Fig. 5:**
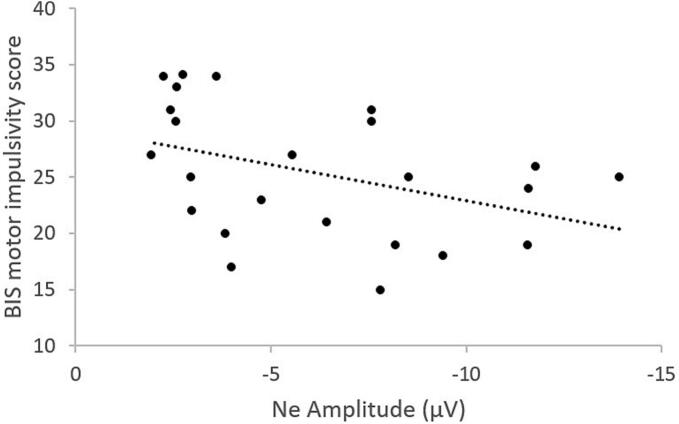
Significant associations within the ADHD group between Ne amplitude at frontocentral sites and the BIS motor impulsivity score*.*

The primary impulsivity scores for each ADHD participant were examined in relation to their respective Ne and Pe components. Analysis revealed a significant negative correlation between Ne amplitude and the BIS motor impulsivity score (Spearman Rho = -0.45, p = 0.016), suggesting that greater Ne amplitude was associated with lower motor impulsivity (Fig. 5). No significant correlation was found for Pe.

## 4 Discussion

ADHD is characterized by impulsivity, hyperactivity, and attentional deficits, leading to the common assumption that affected individuals make errors due to acting without reflection. Clinical evaluations confirm additional difficulties with planning. Given these traits—particularly impulsivity—one might expect individuals with ADHD to exhibit significantly faster RTs than controls, potentially resulting in a higher error rate. While our findings did confirm a higher number of errors in ADHD patients, their RTs were not faster than those of controls, consistent with previous literature (Munger et al., 2022, O'Connell et al., 2009, Wiersema et al., 2009). Instead, our results indicated increased RT variability in the ADHD group, further supporting the widely recognized characteristic of response inconsistency in ADHD (Munger et al., 2022, Salum et al., 2019, Wiersema et al., 2009). This variability may reflect deficits in frontal circuit mechanisms responsible for sustaining attention over extended periods (O'Connell et al., 2009).

Our results indicated a reduced Ne amplitude in the ADHD group compared to the control group, although this finding did not reach statistical significance after adjusting for anxiety, depression and the number of valid trials. While research consistently indicates a reduced Ne amplitude during error monitoring in adults with ADHD (Bellato et al., 2021, Lutz et al., 2021, Marquardt et al., 2018), this attenuation does not always reach levels of statistical significance (Devor et al., 2025, Figuracion et al., 2024, Kaiser et al., 2020, Wiersema et al., 2009). This variability may stem, in part, from limited sample sizes, variations in task design, or inconsistencies in Ne measurement (Clayson, 2020). Moreover, emerging evidence indicates that trait-level emotional dysregulation and transient negative affective states may exacerbate the attenuation of Ne, suggesting that emotional processes play a significant modulatory role in early error monitoring (Balogh et al., 2017, Figuracion et al., 2024, Nunez-Estupinan et al., 2022). The preconscious Ne has been proposed to reflect various cognitive mechanisms, including competition between erroneous and correct response representations (Yeung et al., 2004), mismatch detection between actual and expected responses (Coles et al., 2001), and the processing of outcomes that are less rewarding than anticipated (Holroyd and Coles, 2002).

Our study did not reveal a direct link between Ne amplitude and behavioral performance, aligning with findings by Balogh et al. (2017), who independently reported reduced Ne amplitude and elevated commission error rates in response to negatively valenced stimuli in individuals with ADHD. In contrast, our data indicated that longer Ne latency was significantly related to higher commission error rates and faster response times, suggesting that delayed error monitoring may contribute to impulsive and inaccurate actions.

Based on self-reported clinical assessment, we demonstrated a negative association between Ne amplitude and motor impulsivity, such that individuals with lower Ne amplitudes exhibited higher impulsivity scores. This pattern is consistent with previous evidence linking diminished Ne to heightened impulsiveness, as reflected in fast RTs in healthy participants (Ruchsow et al., 2005). Notably, Taylor et al. (2018) extended this observation to developmental populations, showing that larger Ne amplitudes during adolescence were associated with lower motor impulsiveness. Together, these findings reinforce the notion that both the amplitude and timing of the Ne component are critical markers of individual differences in early error monitoring.

Our results showed a significant reduction in Pe amplitude in the ADHD group compared to the control group, consistent with existing literature (Bellato et al., 2021, Devor et al., 2025, Figuracion et al., 2024, Kaiser et al., 2020, Lutz et al., 2021, Marquardt et al., 2018, Wiersema et al., 2009). Importantly, our finding was observed after adjusting for anxiety, depression and the number of valid trials, which underscores the dominant effect of ADHD on Pe amplitude reduction. Pe is believed to result from conscious cortical integrations that evaluate errors to correct behavior (Bellato et al., 2021, Overbeek et al., 2005, Steinhauser and Yeung, 2010). It is generally larger in trials where participants are consciously aware that they made an error, suggesting that it reflects the motivational significance of an error (Nieuwenhuis et al., 2001, Steinhauser and Yeung, 2010). Hence, its low amplitude would indicate a deficit in error evaluation/awareness (Falkenstein et al., 2000, Kirschner et al., 2021, O'Connell et al., 2009, Ullsperger et al., 2010). Some studies suggest that Pe amplitude is linked to an adaptation, which is reflected in a post-error slowing of response speed to minimize the risk of future commissions (Hajcak et al., 2003, Kirschner et al., 2021, Overbeek et al., 2005). Our results are consistent with this literature, indicating that as the Pe amplitude decreased, the number of commissions increased. Individuals with ADHD exhibited significant RT variability; however, as the Pe amplitude increased, their RTs became less variable and resembled the pattern observed in the control group. ADHD patients exhibited a lower Pe amplitude, which may have contributed to greater difficulty in adjusting their behavior based on past errors, indicating an evaluation deficit. This was associated with a higher number of mistakes and more variable response times, reflecting reduced attentional support (O'Connell et al., 2009).

Several limitations should be considered when interpreting the present findings. One notable constraint is the relatively small sample size in each cohort, which may reduce statistical power and restrict broader applicability of the findings. In particular, the small size of ADHD subgroups based on clinical presentation precludes meaningful statistical analyses of presentation type effects on ERP measures. Another important consideration is the recruitment of ADHD patients from a specialized psychiatric unit, potentially introducing selection bias. This clinical subgroup might reflect more severe manifestations of ADHD than typically seen in broader community samples. Furthermore, medication status varied across participants, and discontinuation was allowed only 24 h before EEG recording. The primary medication used was methylphenidate, which has a relatively short half-life (approximately 2–3 h), making it unlikely to exert residual neurophysiological effects at the time of testing. However, other agents such as atomoxetine and certain neuroleptics have longer half-lives and may not have been fully eliminated. Therefore, the possibility that residual pharmacological or withdrawal-related effects influenced ERP measures could not be completely excluded.

## 5 Conclusions

Our study showed that the Ne and Pe EEG markers of error monitoring can enhance our understanding of the brain processes involved in impulsivity. By associating these markers with both behavioral scores and clinical assessments, we gain a more comprehensive view of the underlying electrophysiological mechanisms. The observed inverse relationship between Ne amplitude and motor impulsivity indicates that individuals with ADHD who present higher impulsivity may exhibit deficits in early error detection. A substantial body of literature links the Ne component to activity in the anterior cingulate cortex (Dhar et al., 2011, Van Veen and Carter, 2002), where individuals with ADHD show reduced gray matter volume (Amico et al., 2011). Moreover, a recent *meta*-analysis identified a cluster within the ACC where reduced volume is significantly correlated with elevated trait impulsivity (Pan et al., 2021), further reinforcing the connection between diminished Ne amplitude and impulsive behavior in ADHD. In parallel, the association between delayed Ne latency and both elevated commission errors and faster RTs points to the importance of timely Ne generation in mitigating impulsive responses. Across the available literature on error-monitoring processes in adult ADHD, the Pe component appears more consistently blunted than the Ne. This observation may stem from several factors: the Pe's larger amplitude, which makes it easier to detect; its presumed multiregional neural generators—including the midfrontal, parietal and posterior insula cortices (Dhar et al., 2011, Orr and Hester, 2012); its lower sensitivity to emotional state variations (Balogh et al., 2017, Figuracion et al., 2024); and its relative insensitivity to age, in contrast to the Ne, reported to change mostly in younger people (Herrmann et al., 2010). The inverse correlation between Pe amplitude and both commission errors and response time variability highlights the importance of consistent activity in the relevant cortical generators for regulating impulsivity and ensuring accurate performance in conscious error monitoring. Future research should focus on ways to modulate these brain responses, whether through cognitive, behavioral, or pharmacological therapeutic approaches, to enhance self-regulation abilities and improve the quality of life for individuals with ADHD.

## Declaration of generative AI and AI-assisted technologies in the writing process

During the preparation of this work the authors used Microsoft Copilot in order to enhance the quality of the English writing. After using this service, the authors reviewed and edited the content as needed and take full responsibility for the content of the publication.

## CRediT authorship contribution statement

**Gabriele Diamanti:** Data curation, Formal analysis, Methodology, Visualization, Writing – original draft, Writing – review & editing. **Julien Colin:** Investigation, Methodology, Software, Writing – review & editing. **Roland Hasler:** Investigation, Methodology, Writing – review & editing. **Tomas Ros:** Conceptualization, Methodology, Software, Writing – review & editing. **Nader Perroud:** Conceptualization, Methodology, Resources, Funding acquisition, Supervision, Writing – review & editing. **Marie-Pierre Deiber:** Conceptualization, Investigation, Formal analysis, Methodology, Resources, Project administration, Writing – review & editing.

## Funding

This work was supported by the Swiss National Center of Competence in Research ‘‘NCCR Synapsy” [grant number 51NF40185897].

## Declaration of competing interest

The authors declare that they have no known competing financial interests or personal relationships that could have appeared to influence the work reported in this paper.
